# Structural and Functional Alterations in the Microbial Community and Immunological Consequences in a Mouse Model of Antibiotic-Induced Dysbiosis

**DOI:** 10.3389/fmicb.2018.01948

**Published:** 2018-08-21

**Authors:** Ying Shi, Lee Kellingray, Qixiao Zhai, Gwenaelle Le Gall, Arjan Narbad, Jianxin Zhao, Hao Zhang, Wei Chen

**Affiliations:** ^1^State Key Laboratory of Food Science and Technology, Jiangnan University, Wuxi, China; ^2^National Engineering Research Center for Functional Food, Wuxi, China; ^3^International Joint Research Laboratory for Probiotics, Jiangnan University, Wuxi, China; ^4^Gut Microbes and Health Programme, Quadram Institute Bioscience, Norwich, United Kingdom; ^5^UK-China Joint Centre on Probiotic Bacteria, Norwich, United Kingdom; ^6^Beijing Innovation Centre of Food Nutrition and Human Health, Beijing Technology and Business University (BTBU), Beijing, China

**Keywords:** gut dysbiosis, ampicillin, microbiota, inflammation, metabolite, mice model

## Abstract

The aim of this study was to establish continuous therapeutic-dose ampicillin (CTDA)-induced dysbiosis in a mouse model, mimicking typical adult exposure, with a view to using this to assess its impact on gut microbiota, intestinal metabolites and host immune responses. Mice were exposed to ampicillin for 14 days and antibiotic-induced dysbiosis was evaluated by alteration of microbiota and gut permeability. The cecal index was increased in the CTDA group, and the gut permeability indicated by fluorescent dextran, endotoxin and D-Lactate in the serum was significantly increased after antibiotic use. The tight-junction proteins ZO-1 and occludin in the colon were reduced to half the control level in CTDA. We found that alpha-diversity was significantly decreased in mice receiving CTDA, and microbial community structure was altered compared with the control. Key taxa were identified as CTDA-specific, and the relative abundance of *Enterococcus* and *Klebsiella* was particularly enriched while *Lachnospiraceae*, *Coprobacillus* and *Dorea* were depleted after antibiotic treatment. In particular, a significant increase in succinate and a reduction in butyrate was detected in CTDA mice, and the triggering of NF-κB enhancement reflected that the host immune response was influenced by ampicillin use. The observed perturbation of the microbiota was accompanied by modulation of inflammatory state; this included increase in interferon-γ and RegIIIγ, and a decrease in secretory IgA in the colon mucosa. This study allowed us to identify the key taxa associated with an ampicillin-induced state of dysbiosis in mice and to characterize the microbial communities via molecular profiling. Thus, this work describes the bacterial ecology of antibiotic exposure model in combination with host physiological characteristics at a detailed level of microbial taxa.

## Introduction

Since their discovery in the early twentieth century, antibiotics have been used widely. In recent decades, antibiotic use has accelerated and been accompanied by extensive public health benefits. However, antibiotics are often used inappropriately; for example, they may be prescribed for short periods to treat flu or acute infections, or added to animal feed as growth promoters (Vigre et al., 2008; Zhou et al., 2013). This overuse and misuse of antibiotics is a worldwide problem, including in China, and there are growing concerns that antibiotic exposure may lead to short-term and/or long-term negative health consequences (Hvistendahl, 2012; Wang et al., 2015).

A well-studied negative impact of antibiotic overuse is the increased development and spread of antibiotic resistance in bacteria. In contrast, the consequences of antibiotic usage on the host’s gut microbial function has received less attention. Gut microbiota play an important role in host metabolism and the immune system (Tremaroli and Backhed, 2012; Caballero and Pamer, 2015). Antibiotics are known to have a significant impact on the composition and functionality of the gut microbiota in humans and mice (Jernberg et al., 2007; Buffie et al., 2012), and thus, perturbation of the microbiota through antibiotic use could be detrimental to host health. Antibiotic-induced alteration of the gut microbiota could also lead to metabolic disorders, such as inflammatory bowel disease, diabetes and obesity (Cho et al., 2012; Boursi et al., 2015; Becattini et al., 2016).

Previous researches have focused on the effects of exposure to combinations of antibiotics on subsequent disease development, particularly asthma, allergies, diabetes and obesity. In our study, we created a murine model of a single antibiotic treatment to explore the consequences of short-term therapeutic-doses of antibiotic on gut microbial ecology and function, and any associated metabolic consequences. We chose ampicillin, a beta-lactam, for this study because it is widely used and has a broad spectrum of activity against various bacterial groups. We used microbial biomarkers based on 16S rRNA gene sequencing data for identification of the microbial groups characteristic of antibiotic-induced dysbiosis and related diseases. Our findings may have implications for antibiotic usage in human clinical practice, routine prescriptions, and their application in livestock.

## Materials and Methods

### Animals and Treatments

Four-week-old male C57BL/6 mice (Slack Experimental Animal Co., Ltd., Shanghai, China) were housed (in pairs) in the Jiangnan University (Wuxi, China) animal facility for one week to acclimatize. Mice were housed in pairs and randomized into two groups (*n* = 8 per treatment group). Continuous therapeutic-dose ampicillin (500 mg/kg, CTDA) was administrated via oral gavage daily for 14 days (CTDA group) while 0.9% saline solution was administrated for 14 days accordingly (Control group). The dose of 500 mg/kg was calculated according to the clinical adult dose and transferred to the experimental mice dose (Nair and Jacob, 2016). All mice had *ad libitum* access to water and chow (Slack Experimental Animal Co., Ltd., Shanghai, China), and the mice and their food intake were weighed every 2 days. Fecal pellets were collected at day 0, 7, and 14 throughout the experiment, the samples collected from CTDA group at day 7 was named CTDA1 and at day 14 was named CTDA2. At the end of the experiment (day 14), all mice from two groups (Control and CTDA2 groups) were sacrificed and the colons, ceca and ilea excised and frozen immediately in liquid nitrogen for further analysis. The experiment was performed under the guidelines of the Ethics Committee of Jiangnan University in China (JN No. 201501671) and every effort was made to minimize animal suffering.

### Microbial Community Analysis

Total genomic DNA was extracted from thawed fecal samples using the FastDNA Spin Kit for Soil (MP Biomedical, United States) and the 16S rRNA gene was amplified with a forward (5′- CCT AYG GGR BGC ASC AG -3′) and reverse (5′- GGA CTA CNN GGG TAT CTA AT -3′) barcoded primer set, targeting the V3-V4 region, as previously described (Zhao et al., 2013). Following purification and quantification of the PCR amplicons, the libraries were prepared using the TruSeq DNA LT Sample Preparation Kit (Illumina, United States) and sequenced on the MiSeq platform (500 cycles paired-end, Illumina). De-multiplexing 16S rRNA gene sequences, quality control, and taxonomic assignment were performed using the open source pipeline Quantitative Insights into Microbial Ecology (QIIME) version 1.9.0. The ChimeraSlayer was used to filter trimmed reads and RDP classifier was used for bacterial taxonomy assignment with a confidence value of 50% and trimmed reads clustered into operational taxonomic units (OTUs) at 97% identity level, and calculated diversity metrics in UniFrac analysis as described by Cox et al. (2014).

#### Comparing Differential Abundance of Taxa

To identify significant differences in the relative abundance of microbial taxa between Control and CTDA mice groups, the linear discriminate analysis (LDA) effect size (LEfSe) algorithm on the Galaxy browser was used (Segata et al., 2011). For LEfSe, the non-parametric Kruskal–Wallis test was used first to identify taxa that were significantly different in abundances followed by LDA to determine the effect size. Taxa were considered to be significantly different to each other if the *P*-value for the factorial Kruskal–Wallis sum rank test was <0.05 and the logarithmic LDA score was > 2.0.

#### Co-occurrence and Diversity of Taxa in Control and CTDA Samples

To identify sets of OTUs that differed amongst the mice intestinal microbiome samples from the Control and CTDA groups, we visualized the co-occurrence network of the samples based on genus level in Cytoscape (version 3.1.0) with an edge-weighted spring-embedded layout. The input files were generated using the QIIME script make_otu_network.py on Control and CTDA fecal samples.

### Visualization of Community Structure Using Non-metric Multidimensional Scaling (NMDS) and Multivariate Analysis

Two dimensional visualizations of the whole community structure were performed using NMDS with Bray–Curtis distances at family or genus levels (Torondel et al., 2016).

Permutational multivariate analysis of variance was used to determine if gut permeability variables had a significant association with microbial community structure. We used the implementation of permutational multivariate analysis of variance (MANOVA) in the Adonis function of the *vegan* package in R. In addition, to determine precisely how the community structure differed between categorical variables, we fitted Dirichlet-multinomial models to the community compositions to each category separately (Holmes et al., 2012). This allowed determination of differences in mean expected taxa abundances and uncertainties in those predictions.

### Identifying Metabolites by ^1^H NMR Analysis

Thawed fecal samples (∼100 mg) were weighed and mixed with saline phosphate buffer as described by Le Gall et al. (2011). Samples were homogenized for 60 s, centrifuged (14,000 × *g*, 15 mins at 4°C), the supernatants filtered through 0.2 μm membrane, and 500 μL of each filtrate transferred to a 5 mm o.d. NMR tube for metabolomic analysis.

^1^H NMR spectra were acquired on a Bruker Avance 600 MHz NMR spectrometer (Bruker, Rheinstetten, Germany). Sample temperature was controlled at 300 K. The *noesypr1d* pre-saturation sequence was used to suppress the residual water signal with low power selective irradiation at the water frequency during the recycle delay (D1 = 2 s) and mixing time (D8 = 0.15 s). Spectra were transformed with 1 Hz line broadening and zero filling, and the baseline corrected using the TOPSPIN 2.0 software. The “underground removal tool” of Bruker AMIX software v3.9 was applied to all spectra to remove the broad irregular envelope which extends from 0.7 to 4.5 ppm. The resulting spectra were divided according to the horizontal axis into variable width “buckets” using the AMIX graphical editor, and the intensities within each bucket were summed.

### Measurement of Cecum Indices

After the sacrifice, the wet weight of each mouse and its cecal tissues were measured. Cecum index values were calculated using wet weight of cecum tissue (g)/mouse weight (g).

### Quantification of Intestinal Permeability (Fluorescent Dextran, Diamine Oxidase, Endotoxin, D-Lactate) in the Serum

Intestinal permeability was measured using 4000 Da fluorescent dextran–FITC (DX-4000–FITC) (Sigma-Aldrich, United States) as described by Cani et al. (2009). On the day 12, after fasting for 6h, mice were administered with DX-4000–FITC (500 mg/kg weight), and blood was collected from the vein at the tip of the tail after 1 h. Serum was diluted (1:1 v/v) in PBS (pH 7.4) and after centrifugation (4,000 × *g*, 10 mins at 4°C) the concentration of DX-4000–FITC was measured using a fluorescence spectrophotometer (Varioskan^TM^ LUX multimode microplate reader, Thermo Fisher Scientific, Finland) at an excitation wavelength of 485 nm and an emission wavelength of 535 nm. Serum sample was taken before sacrifice and the concentrations of diamine oxidase (DAO), endotoxin (ET), and D-lactate determined using Enzyme-Linked Immunosorbent Assay (ELISA) kits (SenBeiJia Biological Technology Co., Ltd., Nanjing, China).

### Measurement of Tight-Junction Proteins in the Colon

To evaluate the integrity of the tight junctions between intestinal epithelial cells, the colon tissues (100 mg) were sectioned and were homogenized in 900 μL PBS using a Scientz-50 mortar-grinder (Lanzhi, Ningbo, China), centrifuged at 13,000 × *g* for 10 min, and the supernatants transferred into sterile tubes. The expression of tight-junction proteins including zonula occludens-1 (ZO-1) and occludin were measured using ELISA kits (SenBeiJia Biological Technology Co., Ltd., Nanjing, China).

### Immunofluorescence Staining of Occludin for Tight Junction Morphology

Sections of ileal tissue were fixed in 4% paraformaldehyde and permeabilized with 0.01% Triton-X prior to incubation with primary occludin rabbit polyclonal antibody (Thermo Fisher Scientific, United States) (1:200 dilution) for 2 h at 37°C. Sections were washed in PBS, and incubated with secondary goat anti-rabbit IgG (1:100 in PBS) (Jackson Immuno Research, United States) for 1 h. Sections were visualized on a fluorescence microscope.

### Measurement of Cytokine Levels in the Colon

The supernatants of colon tissues from Control and CTDA2 groups were obtained using the method described previously in Material and Methods (Expression of tight-junction proteins in the colon). The levels of monocyte chemoattractant protein-1 (MCP-1), interferon-γ (IFN-γ), secretory immunoglobulin (sIgA), nuclear factor kappa-light-chain-enhancer of activated B cells (NF-κB), and regenerating islet derived protein III gamma (RegIIIγ) were determined using specific ELISA kits following the manufacturer’s instructions (Nanjing SenBeiJia Biological Technology Co., Ltd., China).

### Statistical Analysis

Results are presented as means ± SEM. Comparisons between ampicillin-treated mice and Control mice were made using unpaired two-tailed *t*-tests in GraphPad Prism version 7.00 for windows. *P* < 0.05 was considered as a significant level. Statistical differences amongst multiple treatment groups was determined using one-way ANOVA (SPSS 11.0 software).

### Data Deposition

The datasets including raw reads and genome assemblies for this study can be found in NCBI under Sequence Read Archive (SRA) accession number of SRP135803.

## Results

### Ampicillin Administration Alters Colonic Microbial Communities

**FIGURE 1:**
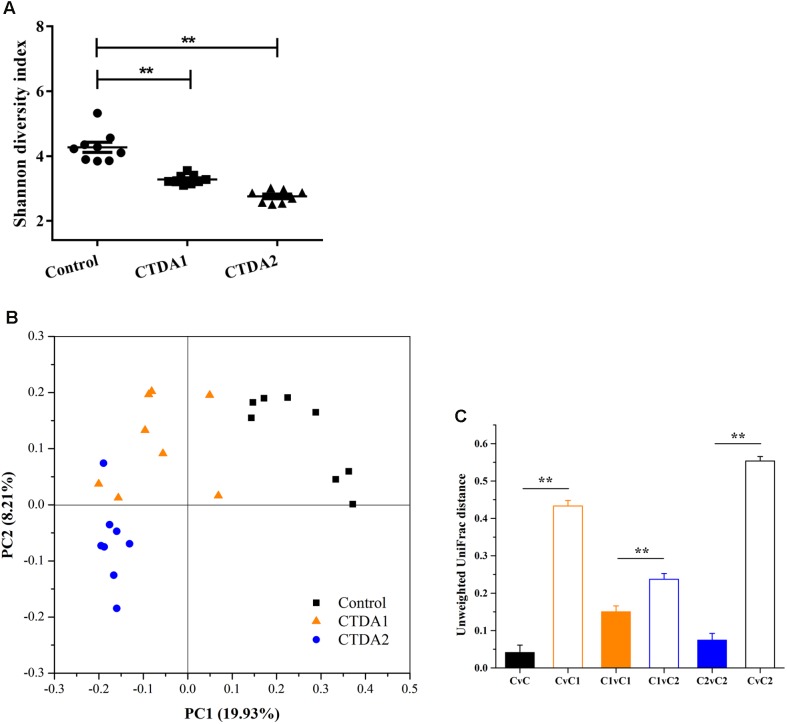
α- and β-diversity in Control, CTDA1, and CTDA2 fecal samples. **(A)** α-diversity as measured by the Shannon diversity index of microbiota in fecal samples from different groups. The CTDA was administrated for 14 days and fecal samples were collected at day 7 (CTDA1 group) and day 14 (CTDA2 group), and the 0.9% saline solution was administrated for 14 days and fecal samples were collected at day 14 (Control group), *n* = 8 per treatment group. The Shannon diversity index for each microbial community was calculated at a depth of 5000 sequences per sample. Two-tailed student’s *t-*test was used to determine statistical significance, ^∗∗^*P* < 0.01**(B)** Visualization of principal coordinates analysis (PCoA) of unweighted UniFrac distances to show differences in bacterial community structure. The first principal component (PC1) and second principal component (PC2) explained 19.93 and 8.21% of the variance in the unweighted UniFrac metrics, respectively. Each point represents the bacterial fecal microbiota in a single sample. **(C)** Pairwise unweighted Unifrac distance measurements of microbiota in fecal samples from Control, CTDA1, and CTDA2 mice. Means ± SEM of the distances are shown. A two-tailed student’s *t*-test was used to determine statistical significance, ^∗∗^*P* < 0.01. C = Control group, C1 = CTDA1 group, C2 = CTDA2 group.

We examined 16S rRNA gene sequences to determine the effects of ampicillin on the diversity of the colonic microbiota. At both time points (day 7 and 14 of ampicillin use), the Shannon diversity values in the CTDA groups were significantly lower than that in the Control (*P* < 0.01) (**Figure 1A**). Quantitative PCR showed that ampicillin significantly reduced the microbial total numbers (*p* < 0.0001; **Supplementary Figure S3**).

After two weeks’ exposure, the fecal microbiota in the CTDA2 group was distinct from the Control group in terms of community structure (β-diversity). Bacterial communities in the one-week samples (CTDA1 group) progressively diverged from the Control group toward the CTDA2 profile (**Figure 1B**). The Unifrac distances between the Control and CTDA groups were significantly greater than intragroup distances (*P* < 0.01) (**Figure 1C**). Overall, these results indicate that CTDA treatment selected for a distinctive microbial community structure, and the diversity and structure of the colonic microbiota among treatment groups and the Control group were more distinct following ampicillin administration over an increased period of time.

### CTDA Affects Microbiota Characteristics Based on Taxa Abundances

**FIGURE 2:**
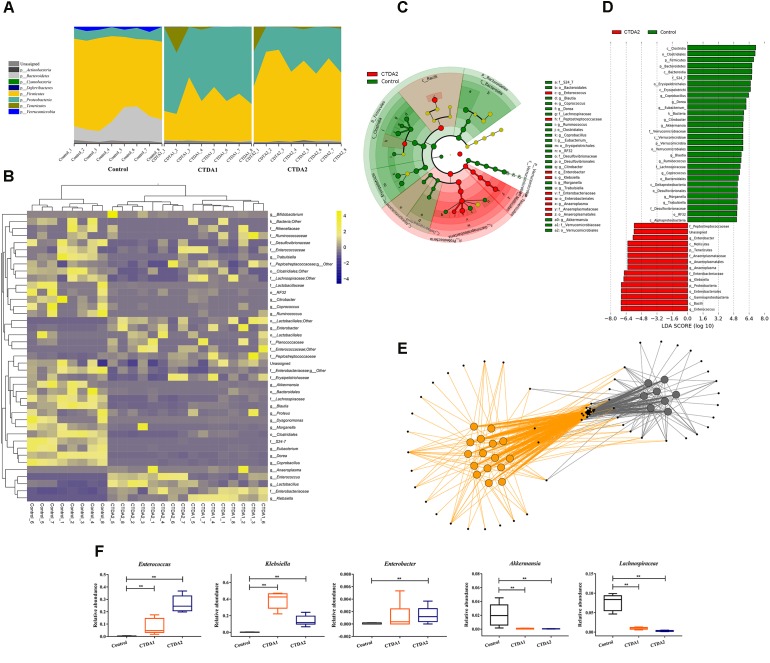
Characteristics of microbiota from Control and CTDA fecal samples. **(A)** Relative abundance of bacterial phyla in fecal samples from Control, CTDA1 and CTDA2 groups of mice. **(B)** Heatmap of differential taxa. Taxonomic Heatmap using Bray-Curtis Dissimilarity index distance, combined with Average (unweighted pair-group method with arithmetic means) clustering for 41 of the most statistically significant OTUs between Control and CTDA samples. Yellow and blue represent high and low abundance, respectively. **(C)** Identification of significant differences in bacterial taxa between CTDA2 and Control group. Cladogram depicting differences and phylogenic location are shown. In each section, the diameter of the circle is proportional to the abundance of the taxon. Fecal microbial communities from CTDA2 and Control mice were compared using LEfSe (green = taxon significantly enriched in Control; red = taxon significantly enriched in CTDA2; yellow = non-significant). **(D)** Histogram of the LDA scores computed for features differentially abundant between the CTDA2 and Control groups. LEfSe scores represent the degree of consistent difference in relative abundance between features in two groups of analyzed microbial communities. The clades of the histogram (red indicating CTDA2 group, green indicating Control group) identifies statistical and biological differences between the communities. **(E)** Taxa co-occurrence network. Cytoscape was used to visualize co-occurrence patterns for CTDA2 and Control mice at the genera level. In the network, samples were colored by treatment group (Control samples = gray; CTDA2 samples = orange). Individual genera are represented by small black nodes. Lines connecting a sample to a genus, colored by treatment group, indicate that the genus was observed in that specific sample. **(F)** Relative abundance of *Enterococcus, Klebsiella, Enterobacter, Akkermansia*, and *Lachnospiraceae* in fecal samples from the CTDA and Control groups of mice. Boxes represent the interquartile range (IQR), median (horizontal line within the box), range (whiskers) and outliers (crosses) (>1.5^∗^IQR). A two-tailed student’s *t*-test was used to determine statistical significance, ^∗∗^*P* < 0.01.

Mice in both CTDA groups showed a complete loss of *Bacteroidetes* and *Verrucomicrobia*, but an increase in the relative proportions of *Proteobacteria* and *Tenericutes* at the phylum level (**Figure 2A**). The taxonomic heatmap (**Figure 2B**), generated using the Bray-Curtis dissimilarity index distance, combined with the average clustering of statistically significant OTUs between Control and CTDA samples, revealed that *Klebsiella* and *Anaeroplasma* were significantly associated with ampicillin use. The relative abundance of *Enterococcus*, *Enterobacter* and *Peptostreptococcaceae* was higher in the two CTDA groups compared with the Control group. Control samples had a significantly higher abundance of *Coprobacillus*, *Dorea*, *Akkermansia*, *Eubacterium*, *Citrobacter*, *Ruminococcus* and *Lachnospiraceae*, which were almost completely lost in both the CTDA1 and CTDA2 samples.

The LDA effect size (LEfSe) was applied specifically to identify taxa that were differentially enriched at genus and family level amongst treatments (**Figures 2C,D**). After modeling, particular taxa including *Klebsiella*, *Anaeroplasma*, *Enterococcus* and *Enterobacter* were over-represented in CTDA2 feces compared with the Control. In contrast, *Lachnospiraceae* and *Akkermansia* were enriched in the Control group rather than the CTDA2 group. Similarly, the relative abundance of *Enterococcus* and *Morganella* increased, while *Eubacterium*, *Blautia*, *Coprococcus*, *Dorea* and *Akkermansia* decreased significantly in the CTDA1 group compared with the Control group (**Supplementary Figures S1**, **S2**). Furthermore, taxa co-occurrence network analysis differentiated the microbiota between the Control and CTDA2 groups; CTDA2 deviated substantially from the Control. Although multiple constituent OTUs overlapped between the two groups, specific genera could be used to distinguish CTDA2 from the Control group (**Figure 2E**).

Focusing on specific microbial changes, CTDA treatment affected multiple taxa, some of which were modulated significantly after exposure. Levels of *Lachnospiraceae* and *Akkermansia* were markedly diminished in the CTDA2 group compared with the Control group (*P* < 0.01) (**Figure 2F**).

### CTDA Affects Levels of Metabolic and Inflammatory Biomarkers

**FIGURE 3:**
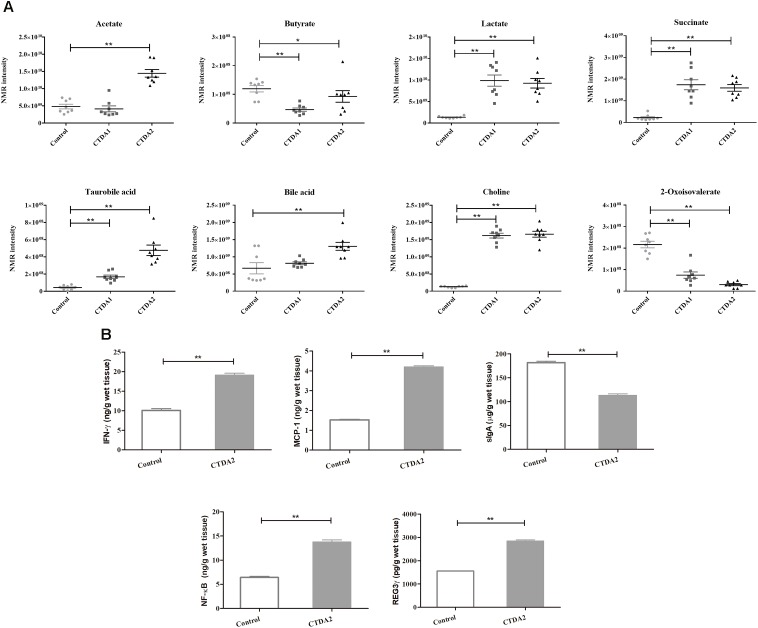
Metabolic and immunological outcomes of the CTDA mouse treatment. **(A)** Distribution of intensities for selected metabolites in the feces from Control and CTDA groups of mice. Horizontal lines show mean and SEM. A two-tailed student’s *t*-test was used to determine statistical significance, ^∗^*P* < 0.05, ^∗∗^*P* < 0.01. **(B)** Levels of inflammatory cytokines measured by ELISA in colonic tissues from Control and CTDA2 groups of mice. A two-tailed student’s *t*-test was used to determine statistical significance, ^∗∗^*P* < 0.01. Error bars represent mean ± SEM (*n* = 8).

Metabolomic analysis of fecal samples showed differences in the profiles of specific metabolites between the Control and the two CTDA groups. The intensity distributions of selected metabolites indicated potentially negative alterations including a consistent decrease in butyrate (*P* < 0.05) and 2-oxoisovalerate, and an increase in succinate, tauro-bile acid, lactate and choline in CTDA2 compared with the Control group (*P* < 0.01) (**Figure 3A**).

Assessment of immunological makers indicated that the levels of IFN-γ, NF-κB, MCP-1 and RegIIIγ were significantly increased in the mice of CTDA2 group compared with the Control group (**Figure 3B**). Production of sIgA was significantly reduced in the CTDA2 group compared with the Control (*P* < 0.01).

Taken together, these results indicate the metabolic alteration and increased intestinal immune responses in two-weeks CTDA treatment of mice.

### Establishment of the Continuous Therapeutic-Dose Ampicillin Mouse Model

**FIGURE 4:**
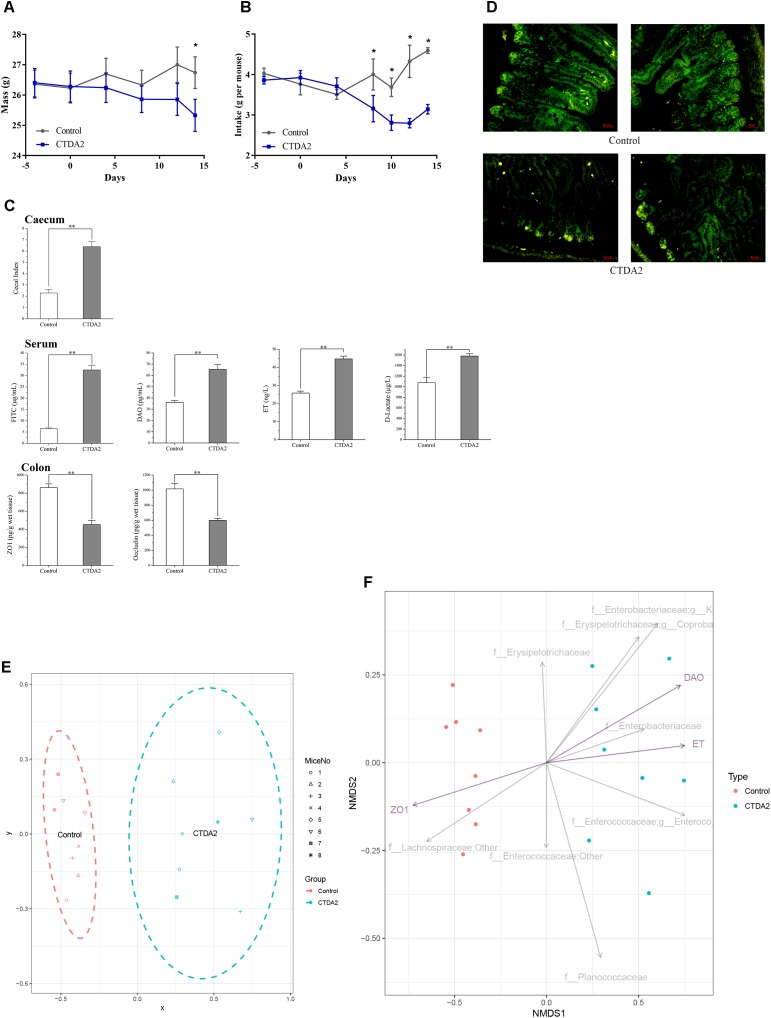
Establishing a CTDA mouse model based on key host biomarkers **(A)** = weight and **(B)** = food intake of mice. Day 4 was the first day of the adaptive phase, ampicillin administration started on day 0, and the end point of ampicillin use was day 14. A two-tailed student’s *t*-test was used to determine statistical significance, ^∗^*P* < 0.05. Error bars represent mean ± SEM (*n* = 8). **(C)** Main factors including cecal indices in cecum, biomarkers associated with the gut barrier isolated from the serum and expression of tight-junction proteins in the colon of Control and CTDA2 mice. A two-tailed student’s *t*-test was used to determine statistical significance, ^∗∗^*P* < 0.01. Error bars represent mean ± SEM (*n* = 8). **(D)** Images representing tight junction disruption (occludin) of the ileum in the Control and CTDA2 groups of mice as determined by immunofluorescence (Representative images, *n* = 4/group). **(E)** Non-parametric monotonic relationship between the dissimilarities in the samples of CTDA2 group (blue, right) and Control group (red, left) matrix. The spots site based on the abundance of microbial taxa, measurement of cecal, colon, and serum samples which listed in **C**. **(F)** Non-metric multidimensional scaling plot of family and genus compositions for the Control (red) and CTDA2 samples (blue) with the three featured variables (purple arrows) (DAO = diamine oxidase, ET = endotoxin, and ZO1 = tight-junction protein ZO-1) plotted using the *EnvFit* function of package in R. The gray arrows give the top eight families or genera that most differ in expected proportion. f_*Enterobacteriaceae*, g_K = f_*Enterobacteriaceae*, g_*Klebsiella*, f_*Erysipelotrichaceae*, g_*Coproba* = f__*Erysipelotrichaceae*, g__*Coprobacillus*, f_*Enterococcaceae*, g_*Enteroco* = f_*Enterococcaceae*, and g_*Enterococcus*.

During the ampicillin administration period, the weight of mice in the CTDA group gradually decreased and was considerably lower than the Control group (*P* < 0.05) on day 14, and the food intake was significantly decreased (*P* < 0.05) in CTDA group after day 7 (**Figures 4A,B**). The CTDA treated mice exhibited significantly higher (*P* < 0.01) cecal index in cecum and DX-4000-FITC in serum compared with the Control mice, and increased levels of DAO, endotoxin and D-lactate were observed in CTDA2 mice. The key markers of tight junction integrity including colonic ZO-1 and occludin were reduced by antibiotic treatment (**Figure 4C**). The examination of occludin stained sections of the ileum confirmed that the levels of this protein were reduced and became discontinuous in mice tissues from CTDA2 group (**Figure 4D**), which was not observed in the Control group.

A range of biomarkers were detected and measured in the CTDA2 mice to establish a model that reflected the status of gut dysbiosis and the impact on the intestinal barrier. The major assessments selected included levels of the cecal index, gut permeability, and expression of tight-junction proteins, that either increased or decreased significantly in the CTDA2 treatment group. Based on the combination of these major biomarkers, we applied a non-metric multidimensional scaling (NMDS) plot of the samples to establish a CTDA model (**Supplementary Table S1**). In this model, the CTDA2 group clearly clustered in an area totally separated from the Control group (**Figure 4E**), implying that CTDA2 group was affected by ampicillin administration and exhibited a profile different from that in the Control group after 2 weeks exposure. In **Figure 4F**, we plotted 8 families or genera with the largest difference in abundance between the two groups. *Klebsiella*, *Coprobacillus*, and *Enterobacteriaceae* were associated with ampicillin-induced microbiota dysbiosis, while *Lachnospiraceae* were more likely to be found in Control group. We also plotted the direction of change in the three significant gut permeability variables (**Figure 4F**). We observed that a host-microbiome correlation exists and the tight-junction protein ZO-1 is negatively associated with specific gut microbiota of antibiotic-induced dysbiosis, while endotoxin and DAO are positively associated with such dysbiosis status.

## Discussion

Numerous studies have reported dysbiosis of the gut microbiome due to administration of a cocktail of antibiotics, however, very few of these have looked at the impact of dysbiosis on the gut barrier integrity or changes in the gut metabolome (Nobel et al., 2015; Mahana et al., 2016). In this study, we established a mouse model that provided CTDA to simulate typical exposure in humans and explored how this single antibiotic modified the gastrointestinal microbial community and its effect on the host immune status and gut barrier function.

### Alteration of Specific Species Could Trigger the Gut Disease

We observed that administration of ampicillin caused a boom in *Proteobacteria*, and reduced the abundance of *Firmicutes* and other phyla. This is consistent with other studies (Thompson et al., 2015; Khan et al., 2016) which indicated a similar increase in the abundance of *Proteobacteria*. This may explain the observed weight loss in CTDA mice, which has also been observed previously where an overabundance of *Proteobacteria* was recorded (Zhang et al., 2009). An increased relative abundance of *Proteobacteria* is also known to lead to a promotion of the transfer of endotoxin and D-lactate across the intestinal barrier, initiating inflammation (Cani et al., 2008). Consistent with a prior study using cefixime (Shi et al., 2017), analysis of β-diversity in the mice feces indicated that CTDA-exposed microbial communities were distinct from the Control group. The relative abundance of *Lachnospiraceae* decreased in both the CTDA1 and CTDA2 groups compared with the Control group. Patients with intestinal dysbiosis were also found to have reduced abundance of *Lachnospiraceae* (Antharam et al., 2013; Pham and Lawley, 2014). Several genera in this family are fibre-degraders and produce short-chain fatty acids (SCFAs). SCFAs enhance the intestinal barrier by regulating the assembly of tight junctions; therefore, decreases in the abundance of *Lachnospiraceae* has been implicated in the leaky gut process. Our data in this study also found that ampicillin-induced dysbiosis resulted in reduction of both levels of SCFAs and abundance of *Lachnospiraceae*.

LEfSe analysis allowed the identification of key characteristics of the microbiota in the CTDA model. As a tool, LEfSe is used to predict which microbial taxa differ significantly between test groups; it emphasizes statistical significance and biological consistency, and identifies differentially abundant features that are consistent with biologically meaningful categories (Segata et al., 2011). Specific OTUs can differ between groups of samples and several features of microbial communities have been identified as potential biomarkers for many disease states (Lecuit and Lortholary, 2005; Manichanh et al., 2006; Oakley et al., 2008). In our study, the dominance of *Klebsiella* following ampicillin exposure is noteworthy because, in humans, *Klebsiella oxytoca* has been identified as the causative organism for antibiotic-associated hemorrhagic colitis (Högenauer et al., 2006). *Klebsiella* also has a key role in the initiation of pathological damage in patients with Crohn’s disease (Rashid et al., 2013). Various studies have suggested that commensal organisms, such as *Enterococcus* and *Klebsiella* species, drive the pathogenesis of experimental intestinal inflammation and human IBD (Sartor, 2006, 2008). The high prevalence of *Klebsiella*, *Enterococcus* and *Enterobacter* in the CTDA2 group, may be the consequence of antibiotic perturbation of the microbiome. The observation that particular species increased in abundance may be due to opportunistic microbes that flourish when ecosystems are disrupted (Garrett et al., 2010). Also, there are studies reported that ampicillin-resistant *Klebsiella* and *Enterococcus* species exist (Klare et al., 2005; Rashid et al., 2012), which could explain the significantly increase in ampicillin group. The decrease in abundance of *Akkermansia* in both CTDA groups is also a negative outcome since *A. muciniphila* has been shown to reduce inflammation in adipose tissues (Shin et al., 2014).

### Succinate and Other SCFAs Indicated the Dysbiosis Status

Notably, we observed that CTDA treatment leads to an increase in levels of succinate in the intestinal lumen; this microbiota-derived nutrient promotes pathogens such as *Clostridium difficile*, and is associated with the development of colitis and cell death (Ferreyra et al., 2014). Lipopolysaccharide (LPS) strongly increased the tricarboxylic acid (TCA) cycle intermediate succinate. While succinate is also involved in adenosine triphosphate generation in the host mitochondria, has a signaling capacity; and is identified as a metabolite in innate immune signaling which leads to enhanced IL-1β production via HIF-1α during inflammation (Tannahill et al., 2013). Moreover, the LPS endotoxin stimulation in macrophages is associated with an increase in mitochondrial citrate carrier (CIC), possibly due to activation of the NF-κB sites contained in the CIC promoter (Infantino et al., 2011). The increased levels of endotoxin in serum, and NF-κB in the colon, that we observed following CTDA treatment also supported the fact that LPS induced the activation of NF-κB pathway. We also observed elevated levels of IFN-γ in CTDA mice, this was also demonstrated previously which related to antigen-specific T cell activation when dendritic cells (DCs) were primed simultaneously with succinate and antigen (Rubic et al., 2008).

Acetate, propionate, and butyrate are the main SCFAs produced during fermentation by the gut microbiota. In this study, we found that fecal butyrate was reduced in both CTDA1 and CTDA2 groups while acetate and lactate increased in the CTDA2 group compared with the Control. Butyrate is a major energy source for intestinal epithelial cells, and is also believed to have anti-inflammatory properties and the ability to enhance epithelial antibacterial barrier defenses. Defects in butyrate utilization by epithelial cells is thought to be characteristic of antibiotic perturbation and IBD (Dethlefsen and Relman, 2011; Kostic et al., 2014). Increased levels of lactate have been measured in feces from patients with ulcerative colitis (Hove et al., 1994). The levels of tauro-bile acid were significantly higher in both CTDA1 and CTDA2 groups compared with the Control, while levels of bile acid only slightly increased in the CTDA2 group. Tauro-bile acids are a tauro-conjugated bile acid from a subgroup in the bile acids and generally include taurocholic acid, taurodeoxycholic acid, tauro-beta-muricholic acid, and tauro-omega-muricholic acid, which are the main forms found in the fecal samples of mice. Taurine may be present in fecal water as a result of bacterial deconjugation of bile acids (Ridlon et al., 2006). Enterococci are known to produce bile salt hydrolases (BSH) that convert conjugated bile acids into free bile acids (Jiao et al., 2017) and we speculate whether the increase in free bile acid is a reflection of observed increase in abundance of *Enterococcus*. Also, an increase in primary bile acids in the feces of diarrhea-predominant IBS (IBS-D) patients has been detected and this was correlated with dysbiosis of bacterial groups (Duboc et al., 2012).

### Microbe-Associated Ecological Disturbances Led to a Leaky Gut

Increased gut permeability could trigger systemic inflammation due to the movement of microbial elements across the epithelial barrier, which is a known marker for intestinal health. Our data suggest that the intestinal barrier was substantially compromised in the ampicillin-induced dysbiosis model, indicated by decreased ZO-1 and occludin levels in the colon. Meanwhile, the increased levels of circulating 4 kDa FITC-dextran molecules detected indicates a weakening of intestinal integrity following ampicillin use. Indeed, translocation of pro-inflammatory mediators, including endotoxin and D-lactate produced by gram-negative enterobacteria, into the systemic circulation (a phenomenon known as “leaky gut”) may play a role in antibiotic-induced dysbiosis. Microbiota changes were also accompanied by an increase in inflammatory activity in the colon and serum. Microbe-associated molecular patterns (MAMPS) can be sensed by intestinal epithelial cells, and induce numerous effects including tissue repair and the production of proteins such as RegIIIγ (a bactericidal C-type lectin) in the intestinal epithelial. Microbiota-derived LPS maintains basal level expression of RegIIIγ in intestinal epithelial cells; RegIIIγ is not detected in germ-free mice and was impaired by short-term antibiotic treatments evaluated in previous studies (Cash et al., 2006; Brandl et al., 2008). Consistent with increasing levels of circulating endotoxin, the expression of RegIIIγ is consequently increased, which is linked to the proliferation of intestinal macrophages (Yoshiok et al., 2009). The composition of CD4^+^ T cells within the intestinal mucosa, DC migration and IL-1β production had been shown to be impaired in antibiotic-treated mice (Ichinohe et al., 2011; Hooper et al., 2012). Our results are also in agreement with the study of Yoshiok et al. (2009), which used β-lactam antibiotics to enhance the development of a systemic inflammatory response. This is achieved through stimulation of endothelial cells with the consequence that granulocytes are enhanced and quantities of MCP-1 are secreted (van Langevelde et al., 1999).

By profiling the microbial 16S rRNA gene, we identified key members of the microbiota that were impacted by either antibiotic exposure or corresponded host physiological changes. If humans have analogous bacterial species, their loss due to antibiotic effects may have important consequences for host health. Furthermore, humans tend to consume a more varied diet and are exposed to a wider range of antibiotics than experimental mice, which may introduce factors that influence recovery from metabolic disruption. However, the development of animal models provides insights into the potential impact of antibiotics on microbiota and how this may relate to metabolic and immunological changes, which may be mirrored in humans. Based on this research, further studies are needed to evaluate the interactions between specific metabolites and key members of the gut microbiota, and to examine whether these effects are similar in humans.

## Conclusion

In conclusion, treatment with typical dose of ampicillin affects the diversity and structure on the gut microbiota, and the differentially abundant features were detected using LEfSe algorithm. The NMDS ecological profiling characterized the microbiota in the ampicillin-induced dysbiosis model, and revealed the specific alterations of bacterial communities were associated to the endotoxin level and expression of tight-junction protein ZO-1. In addition, ampicillin-induced dysbiosis affects the metabolome of mice, and significantly impacts cecal magnitude, gut permeability, and colonic immunity. Based on the determination of gut microbiota composition, metabolites, and immunological barrier, we have developed a model to explore the impact of antibiotic on host factors, and this model can be used to test the effect of intervention strategies aimed at restoring antibiotic induced damage.

## Author Contributions

YS, QZ, and HZ designed the experiments. YS carried out the experiments and wrote the manuscript. GLG analyzed the NMR experimental results. YS and LK analyzed the sequencing data and developed the analysis tools. JZ and AN assisted with the Illumina sequencing. AN and WC revised the manuscript.

## Conflict of Interest Statement

The authors declare that the research was conducted in the absence of any commercial or financial relationships that could be construed as a potential conflict of interest.
