# Supplementary material for: Structural and Functional Alterations in the Microbial Community and Immunological Consequences in a Mouse Model of Antibiotic-Induced Dysbiosis
Source: Front Microbiol. 2018 Aug 21;9:1948. doi: 10.3389/fmicb.2018.01948 (PMC6110884; doi:10.3389/fmicb.2018.01948)
Supplement: Supplementary file 1 [file Image_1.PDF]

frontiers

Supplementary Material

Structural and functional alterations in the microbial community and immunological consequences in mice model of antibiotic-induced dysbiosis

Ying Shi, Lee Kellingray, Qixiao Zhai*, Gwenaelle Le Gall, Arjan Narbad*, Jianxin Zhao, Hao Zhang, Wei Chen

*** Correspondence:** Qixiao Zhai: zhaiqixiao@sina.com; Arjan Narbad: arjan.narbad@quadram.ac.uk

1 Supplementary Figures and Tables

1.1 Supplementary Figures

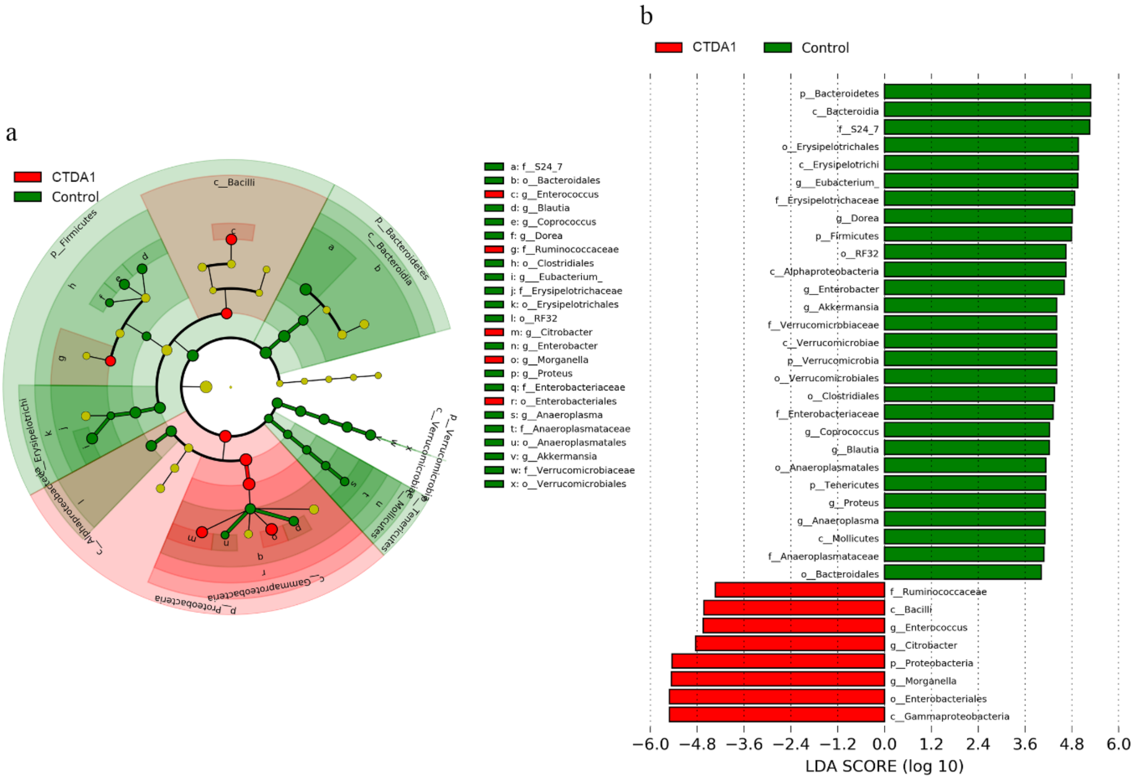

Supplementary Figure 1. Comparison between Control and CTDA1 mice highlighting that, at the phylum level, Proteobacteria are enriched in CTDA1 mice, whereas Firmicutes are enriched in control mice

a. Taxonomic identification of significant differences between CTDA1 and the Control group. Cladograms depict differences and taxonomic level. In each panel, each circle's diameter is

proportional to the taxon's abundance. Faecal microbial communities from CTDA1 and Control mice were compared using LEfSe (green = taxon significantly enriched in control; red = taxon significantly enriched in CTDA1; yellow = non-significant).
b. Histogram of LDA scores computed for features that have differential abundance in CTDA1 and Control groups of mice. LEfSe scores represent the degree of consistent difference in relative abundance between features in the two groups of analysed microbial communities. The clades of histogram (red indicating the CTDA1 group, and green indicating the Control group) identifies statistical and biological differences between communities.

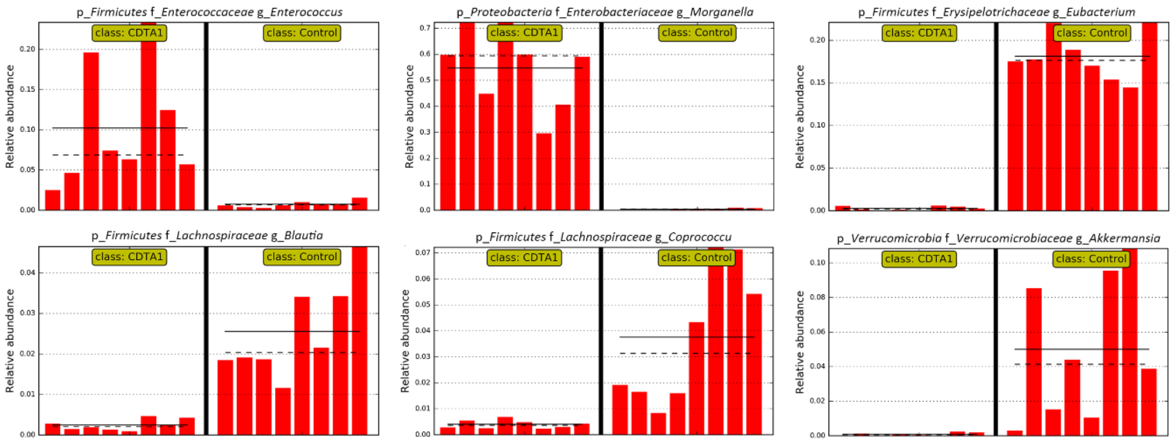

Supplementary Figure 2. LEfSe analysis highlighting bacterial microbiomes at the genus level that are consistently different between the CTDA1 and Control groups of mice. Using the Kruskal-Wallis test portion of LEfSe (α = 0.05), our results showed that six genera differed between the two groups.

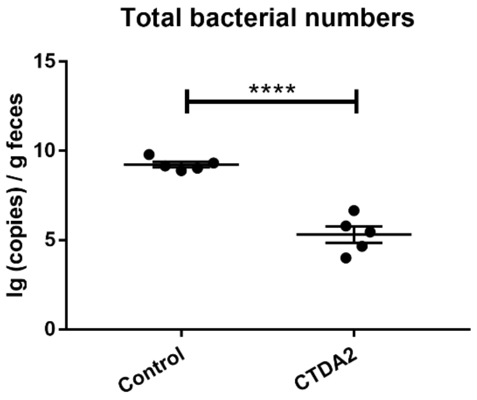

Supplementary Figure 3. Total bacterial numbers in Control and CTDA2 samples. P values are based on unpaired t test, with statistical significance being defined **** as p < 0.0001.

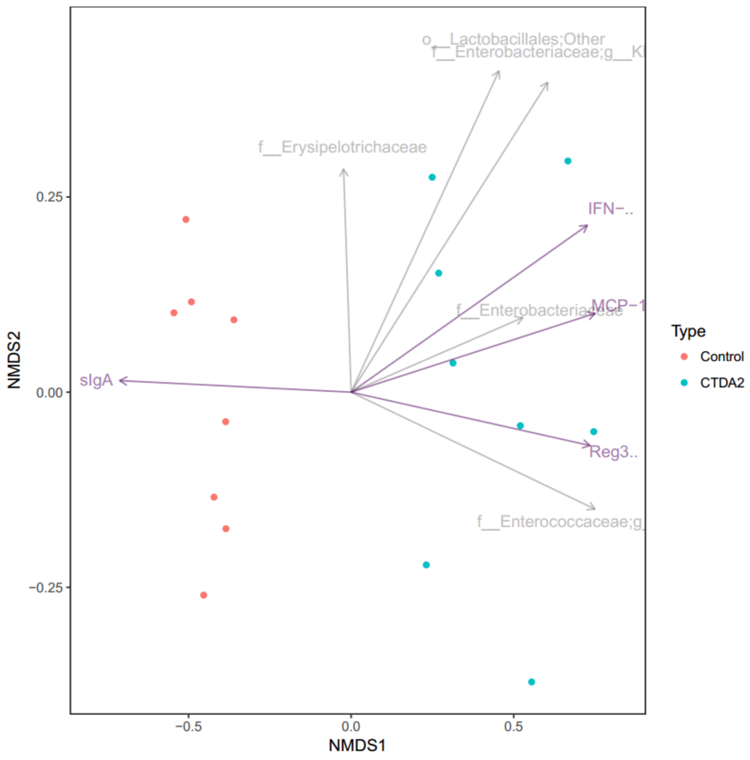

Supplementary Figure 4. Non-metric multidimensional scaling plot of family and genus compositions for the Control (red) and CTDA2 samples (blue) with the four featured immune variables (purple arrows) (IFN = IFN-γ, MCP-1, Reg3 = Reg3γ and sIgA) plotted using the EnvFit function of package in R. The grey arrows give the top 5 families or genera that most differ in expected proportion.

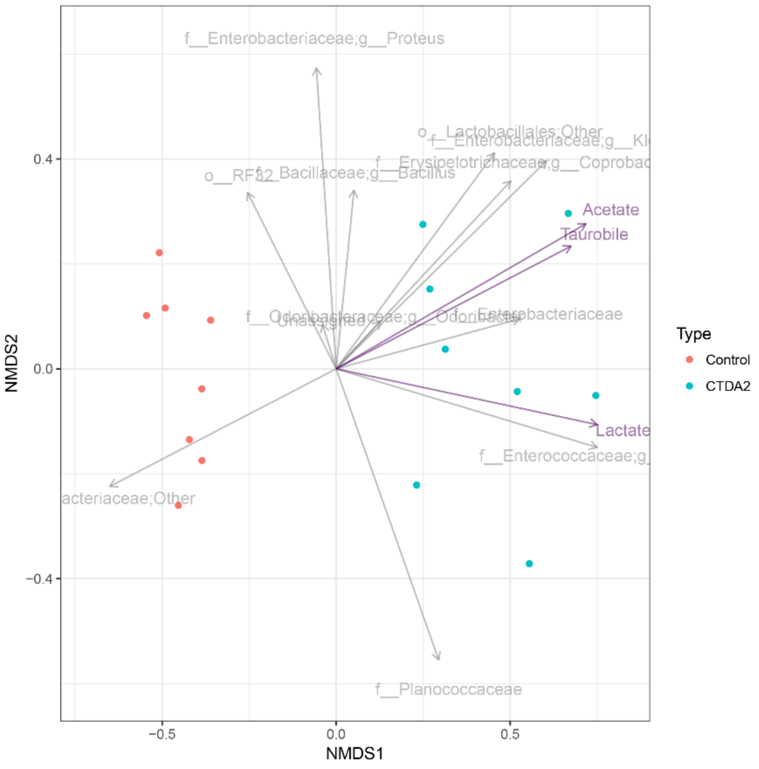

Supplementary Figure 5. Non-metric multidimensional scaling plot of family and genus compositions for the Control (red) and CTDA2 samples (blue) with the three featured metabolites (purple arrows) (Acetate, Taurobile and Lactate) plotted using the EnvFit function of package in R. The grey arrows give the top 15 families or genera that most differ in expected proportion.
